# Effect of Glass Fibers on the Mechanical and Transport Properties of Polymer Inclusion Membranes Composed of Aliquat 336 and PVDF-HFP

**DOI:** 10.3390/membranes16040141

**Published:** 2026-04-01

**Authors:** Lea Kukoc, Kalina Velikova, Sanja Perinovic-Jozic, Maja Biocic, Milen Gateshki, Spas D. Kolev, Tony G. Spassov

**Affiliations:** 1 Faculty of Chemistry and Technology, University of Split, Rudjera Boskovica 35, 21000 Split, Croatia; 2 Faculty of Chemistry and Pharmacy, Sofia University “St. Kl. Ohridski”, 1 J. Bourchier Blvd., 1164 Sofia, Bulgaria; 3 Malvern Panalytical B.V., Lelyweg 1, 7602 EA Almelo, The Netherlands; 4 School of Chemistry, The University of Melbourne, Parkvillee, VIC 3010, Australia; 5 Department of Chemical Engineering, The University of Melbourne, Parkville, VIC 3010, Australia; 6 National Centre of Excellence Mechatronics and Clean Technologies, 8 Kliment Ohridski Blvd., 1756 Sofia, Bulgaria

**Keywords:** polymer inclusion membrane, glass fibers, Aliquat 336, PVDF-HFP, mechanical properties, thermal properties, thiocyanate transport

## Abstract

Polymer inclusion membranes (PIMs) based on PVDF-HFP as the base polymer and Aliquat 336 as the carrier in a mass ratio of 6:4 with concentrations of embedded glass fibers up to 5 wt% were successfully fabricated. Their microstructure, as well as their mechanical and thermal properties, were characterized using scanning electron microscopy (SEM), small-angle X-ray scattering (SAXS), differential thermal analysis/thermogravimetric analysis (DTA/TGA), and tensile testing. Membrane performance and long-term stability in transporting thiocyanate ions were evaluated in a two-compartment transport cell. The results showed that the membranes retained their amorphous structure even with glass-fiber loadings of up to 5 wt%. The addition of glass fibers was found to primarily enhance the elastic modulus and tensile strength, while causing a moderate reduction in plasticity without negatively affecting membrane transport properties and long-term stability. Therefore, it was concluded that the incorporation of glass fibers could improve the suitability of PIMs for industrial applications.

## 1. Introduction

Separation based on the use of liquid membranes has developed as an attractive “green” alternative to conventional solvent extraction (SX) by minimizing or eliminating the use of solvents which are often hazardous (i.e., flammable, toxic and volatile) [1]. Since their introduction, polymer inclusion membranes (PIMs) have been successfully applied for the separation of a wide range of chemical species, including metal ions [2,3,4,5,6], small organic molecules [7,8,9,10,11], and inorganic anions [12,13,14,15]. Therefore, PIMs have shown significant potential for applications in a number of areas such as water clean-up (e.g., mining waters [13,16,17], radioactive waste water [18,19,20,21], municipal and hospital wastewaters [22,23,24], metal plating wastewater [25,26,27,28], agricultural waters [29,30,31,32]); hydrometallurgical separation involving the recovery of Pt group metals [33,34,35,36], rare earth metals [37,38,39,40] and lithium [41,42,43,44], among others; and chemical analysis with a focus on environmental water monitoring and analysis [45,46,47,48]. PIMs have also shown their suitability for continuous separation processes in industry [13] where they separate streams of feed and receiving solutions; they are also applied in online separation in chemical analysis [45,49,50].

PIMs typically consist of a base polymer—most commonly poly(vinyl chloride) (PVC), cellulose triacetate (CTA) or poly(vinylidene fluoride) (PVDF) derivatives such PVDF-hexafluoropropylene (PVDF-HFP), an extractant (often referred to as carrier), and in some cases they may incorporate a plasticizer or modifier [1,4]. The carrier, which is usually an extractant used in conventional SX (e.g., Aliquat 336, di(2-ethylhexyl)phosphoric acid (D2EHPA), Cyanex^®^ reagents), is in most cases a complexing, solvating or ion-exchange reagent which binds the target chemical species at the membrane/feed solution interface, thus facilitating its extraction into the PIM [1]. The carrier usually acts as a plasticizer, but in some cases a separate plasticizer (e.g., dioctyl phthalate, 2-nitrophenyl octyl ether) might be required for ensuring the compatibility between the base polymer and the carrier, while occasionally a modifier (e.g., dodecanol, tetradecanol) may be needed to improve the solubility of the extracted target chemical species-carrier adduct in the PIM liquid phase consisting of the non-polymeric PIM components (i.e., carrier, plasticizer and modifier) [1]. The base polymer incorporates the PIM liquid phase within its entangled chain structure [51], thus acting as the PIM scaffold and ensuring the membrane’s mechanical strength and structural integrity [1,52].

The long-term stability of PIMs is an important characteristic which determines their industrial utility. It depends on their mechanical strength combined with relatively good plasticity and the rate of loss of the PIM carrier due to leaching into the adjacent aqueous phases (i.e., feed and receiving solutions) separated by the membrane.

The leachability of the carrier depends on its aqueous solubility on one hand and, on the other, on its physicochemical interactions with the base polymer, which can decrease leachability, as demonstrated in a recent study by Velikova et al. [53].

The mechanical strength and plasticity of a PIM, characterized by a relatively higher elastic moduli and tensile strength and allowing it to resist tearing or deforming under mechanical stress, is determined by a combination of intermolecular forces and the process of entanglement with polar interactions often resulting in a rigid polymer structure [54]. Polymers with high chemical resistance, higher mechanical strength, and excellent thermal stability such as PVDF [55] and its derivatives (e.g., PVDF-HFP [56,57,58,59]) have gained popularity as base polymers for PIMs. In some cases they have also significantly improved PIM permeability compared to PIMs incorporating other base polymers such as PVC or CTA, for example; this includes the transport of thiocyanate with Aliquat 336 as the PIM carrier [56]. Other approaches for improving the long-term stability of PIMs include the incorporation into the PIM structure of a semi-interpenetrating crosslinked polymer network (e.g., poly(ethylene glycol) dimethacrylate [60]) or different nanomaterials, such as ferrite Fe_3_O_4_, SiO_2_, TiO_2_, multiwalled carbon nanotubes [61] and reduced graphene oxide [62]. However, the majority of these studies mainly investigated the reduction in carrier leachability rather than the improvement in PIM mechanical properties.

PIMs containing Aliquat 336 as the carrier and base polymers such as PVC and PVDF-HFP have been extensively studied for the transport of thiocyanate with the aim of developing a PIM-based method for the clean-up of this toxic anion from gold mine tailings waters and groundwater near gold mines [13,60,63]. These studies have demonstrated that PVDF-HFP is the more suitable base polymer than PVC for this purpose and that the best performance of a PIM composed of Aliquat 336 and PVDF-HFP in terms of PIM extraction capacity and rate of membrane transport for thiocyanate can be achieved when the PIM contains 40 wt% Aliquat 336 [56,60]. Therefore, it was decided to utilize PIMs with a mass ratio of Aliquat 336 to PVDF-HFP of 4 to 6 to illustrate the potential of glass fibers when incorporated into the PIM structure to improve PIM mechanical properties.

In the present study, short glass fibers (GFs) were incorporated at varying concentratio ns into the abovementioned PIMs to enhance their mechanical strength. The resulting polymer inclusion glass fiber membranes were characterized with respect to both their mechanical properties and transport performance using thiocyanate as a model target chemical species.

## 2. Materials and Methods

### 2.1. Solution and Membrane Preparation

The following reagents used in this study were purchased from Sigma-Aldrich (St. Louis, MO, USA): Aliquat 336 (mixture of quaternary alkylammonium chlorides with the dominant species being N-methyl-N,N,N-trioctyl ammonium chloride), PVDF-HFP (average Mw ~ 400,000, average Mn ~ 130,000), tetrahydrofuran (THF, inhibitor-free), Fe(NO_3_)_3_.9H_2_O, NaSCN, HNO_3_ (ACS reagent, 70%) and NaNO_3_ (ACS reagent, 99%). Solutions of 2.0 mM NaSCN used as the feed solution and 1.0 M NaNO_3_ used as the receiving solution in the transport experiments were prepared with deionized water (18 MΩ·cm, Millipore Synergy 185 system, Millipore, Burlington, MA, USA), while a solution containing 0.1 M Fe(NO_3_)_3_ used for the determination of the SCN^−^ concentration was prepared in 0.5 M HNO_3_. GFs with an average diameter of 11.0 ± 1.1 μm were purchased from Glaswarenfabrik Karl Hecht (Sondheim, Germany) and cut to a length between 4 and 6 mm.

**Figure 1:**
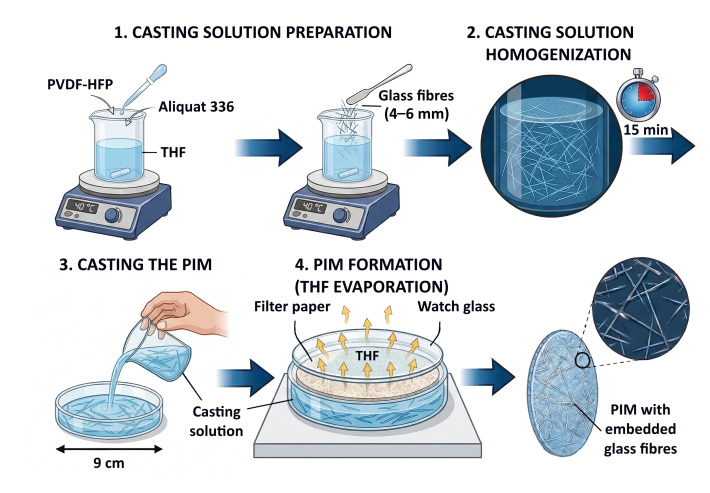
Schematic of the fabrication of PIMs containing PVDF-HFP: Aliquat 336 in a mass ratio of 6:4 and glass fiber concentrations between 1 and 7%.

All % concentrations used in this article are mass % concentrations. Conventional PIMs containing 60% PVDF-HFP and 40% Aliquat 336 and no GFs (PIM-0%GF) were prepared by evaporative casting [63]. A PIM casting solution of 240 mg of the polymer and 160 mg of Aliquat 336 in 15 mL of THF, prepared by heating at 40 °C and stirring on a mixing platform (MULTI-HS 6/15 Digital Multi-position Hot Plate Stirrer, VELP, Usmate, Italy), was used for the fabrication of each membrane. After the membrane components were completely dissolved, the solution was poured into a glass Petri dish (90 mm in diameter) and covered with filter paper and a watch glass to retard solvent evaporation. It was then left to stand for 24 h to allow complete solvent removal. PIMs containing GFs between 1 and 7% (PIM-1%GF–PIM-7%GF) were prepared in the same way as the conventional PIMs (PIM-0%GF) but in this case the relevant amount of GFs were added to the corresponding casting solutions, which were stirred for 15 min to achieve satisfactory solution homogenization based on visual observation, prior to pouring them into glass Petri dishes (Figure 1). All PIMs were conditioned for 24 h in 1 M NaNO_3_ solution prior to their use in the transport experiments to allow the complete exchange of the chloride anion of Aliquat 336 (NR_4_^+^Cl^−^) with a nitrate anion (NR_4_^+^NO_3_^−^ (Equation (1)).

(1) $${\left[{NO}_{3}^{-}\right]}_{aq}+{\left[{\left(NR_{4}\right)}^{+}{Cl}^{-}\right]}_{PIM}\to {\left[{\left(NR_{4}\right)}^{+}{NO}_{3}^{-}\right]}_{PIM}+{\left[{Cl}^{-}\right]}_{aq}$$

### 2.2. Membrane Transport and Stability Studies

The thiocyanate transport studies were carried out in a transport cell consisting of two compartments containing 120 mL of feed or receiving solutions with a PIM of 50 mm in diameter (19.6 cm^2^ exposed to the solutions area) sandwiched between them. The solutions were stirred at 600 rpm. Each extraction experiment included two solutions—a feed solution and a receiving solution, separated by the membrane under investigation. Samples (0.5 mL) were collected from both solutions at predetermined times and replaced with the same volume of the original feed or receiving solution. Each sample was diluted to 3.0 mL with deionized water and mixed with 1 mL 0.5 M HNO_3_ and 0.5 mL 0.1 M Fe(NO_3_)_3_ to form the red-colored [Fe(H_2_O)_5_SCN]^2+^ complex, the concentration of which was determined spectrophotometrically at 460 nm (Shimadzu UV-1601 UV-VIS spectrometer, Shimadzu, Kyoto, Japan).

The retention of Aliquat 336 in PIMs with GF concentrations between 0 and 5% was compared. The corresponding Aliquat 336 batch leaching experiments involved the determination of the mass loss of membranes, with each immersed consecutively in four 120 mL solutions containing 0.1 M NaNO_3_. The immersion time in each solution was 6 h and each solution was stirred at 600 rpm using a magnetic stirring bar.

The long-term stability of PIM-3%GF, which provided an acceptable compromise between strength and plasticity, was compared to that of the conventional PIM-0%GF in 4 consecutive transport experiments with fresh feed and receiving solutions used in each one of them. The mass of each of the two PIMs was measured after each transport experiment to assess the loss of Aliquat 336.

### 2.3. Microstructure and Thermal Analyses

A scanning electron microscope (SEM, Hitachi, Tokyo, Japan) was used to characterize the microstructure of the PIMs with GFs. Small-angle X-ray scattering (SAXS) measurements of these PIMs were conducted on a laboratory X-ray scattering instrument equipped with a ScatterX^78^ evacuated chamber, an X-ray tube with a Cu anode, a focusing multilayer mirror, and a PIXcel^3D^ detector (Empyrean Nano Edition, Malvern Panalytical B.V., Almelo, The Netherlands). To verify the homogeneity of the membranes, four pieces were cut from different locations of each studied membrane. Multiple datasets were collected, including background scans and scans of a conventional PIM-0%GF.

Thermal analyses (thermogravimetric analysis (TGA) and differential thermal analysis (DTA)) of the membranes were conducted on a simultaneous thermal analyzer (SDT650, TA, New Castle, DE, USA) to study their thermal stability and degradation. The thermal methods were also used to assess the stability of Aliquat 336 within the polymer matrix, providing insight into potential decomposition temperatures and Aliquat 336 retention at elevated temperatures. The thermal behavior of the membranes was investigated at 10 K/min under pure nitrogen atmosphere.

### 2.4. Mechanical Properties

Mechanical testing was conducted on a tensile tester (Z020, ZwickRoell, Ulm, Germany) with a 20 kN load cell. Dumb-bell-shaped PIM specimens (length of 55 mm and width of the central section of 12 mm) were tested. The thickness of the central section of each specimen was measured with a digital caliper and care was taken to ensure that the tested PIM specimens were free from surface defects. All tests were carried out at room temperature (25 ± 1 °C) and at a crosshead speed of 1 mm min^−1^ for tensile modulus and 5 mm min^−1^ as the test speed. For each PIM composition, five specimens were tested and the mean values of Young’s modulus (*E*), elongation at break (*ε_B_*) and tensile strength (*σ_B_*) were calculated.

## 3. Results and Discussion

### 3.1. Morphological and Thermal Properties of the PIMs

PIMs were prepared with GFs in the range from 0 to 7% and the mass ratio of PVDF-HFP:Aliquat 336 was 6:4. The GFs were found to distribute relatively uniformly within the PIM structure in concentrations up to 5%, while at higher concentrations they formed aggregates; therefore, only PIMs with GF concentrations up to 5% were studied further.

**Figure 2:**
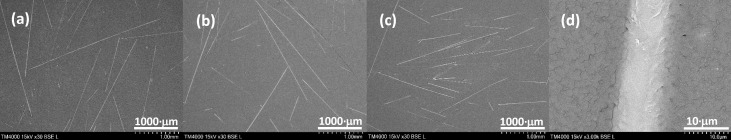
SEM micrographs of the surface of PIM-1%GF (**a**), PIM-2%GF (**b**) and PIM-5%GF (**c**), and higher magnification micrographs of a GF in a PIM matrix (**d**). All PIMs contained PVDF-HFP:Aliquat 336 mass ratio of 6:4.

One of the key factors in analyzing the influence of embedded GFs on the mechanical and transport properties of PVDF-HFP-based PIMs is their distribution throughout the membrane volume. SEM micrographs of PIM-1%GF–PIM-5%GF (Figure 2a–c) show that even on the microscale level the GFs are relatively evenly distributed but randomly oriented in all directions, without a preferred alignment. Another critical microstructural characteristic of the PIMs studied, which is illustrated in Figure 2d, is the good adhesion of the GFs to the PVDF-HFP/Aliquat 336 part of the PIMs, which is expected to have a direct impact on the membranes’ mechanical properties. This good adhesion is facilitated by the uneven surface of the GFs.

**Figure 3:**
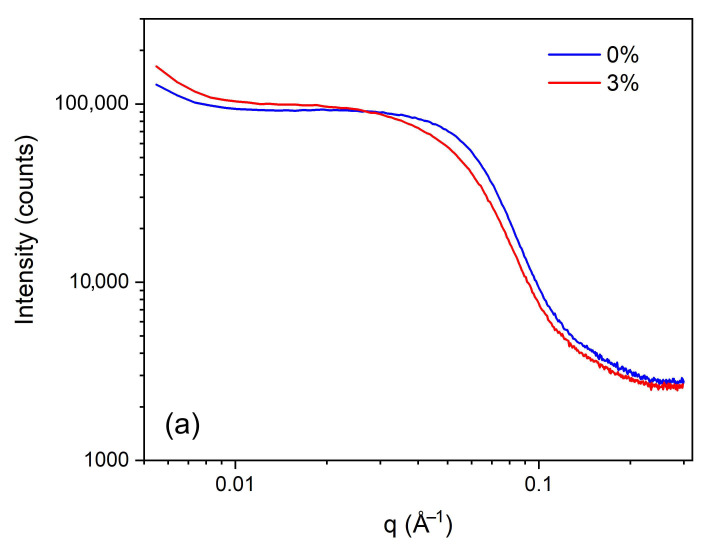

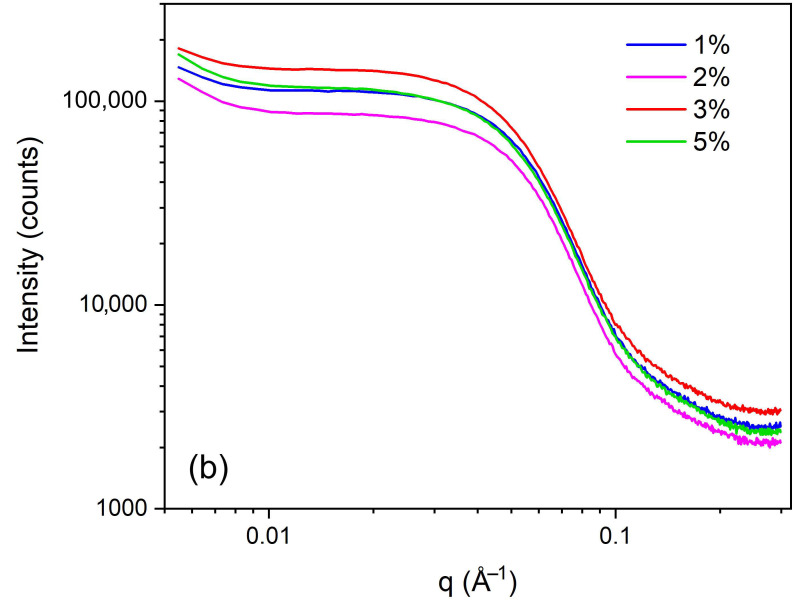
SAXS patterns of PIM-0%GF and PIM-3%GF (**a**), and of PIMs with 1, 2, 3, and 5% GFs (**b**). All PIMs contained PVDF-HFP:Aliquat 336 mass ratio of 6:4.

SAXS analyses indicated that both the membrane without GFs and the GF-reinforced membranes were predominantly amorphous, as illustrated with the diffractograms of the conventional PIM-0%GF and PIM-3%GF, which provided an acceptable compromise between strength and plasticity (Figure 3a). No variations were detected in the diffractograms collected from different sites within the same membrane. No significant trend could be found in the variation in the shape of the signal with respect to the concentration of GFs in the range of 1–5% (Figure 3b). Differences between membranes with varying GF concentrations appeared minimal and were limited to slight changes in the characteristic length scale of the electron density fluctuations. In PIM-0%GF, these regions measured approximately 3.3 nm, whereas in the GF-containing membranes they were slightly larger, i.e., around 3.6 nm.

**Figure 4:**
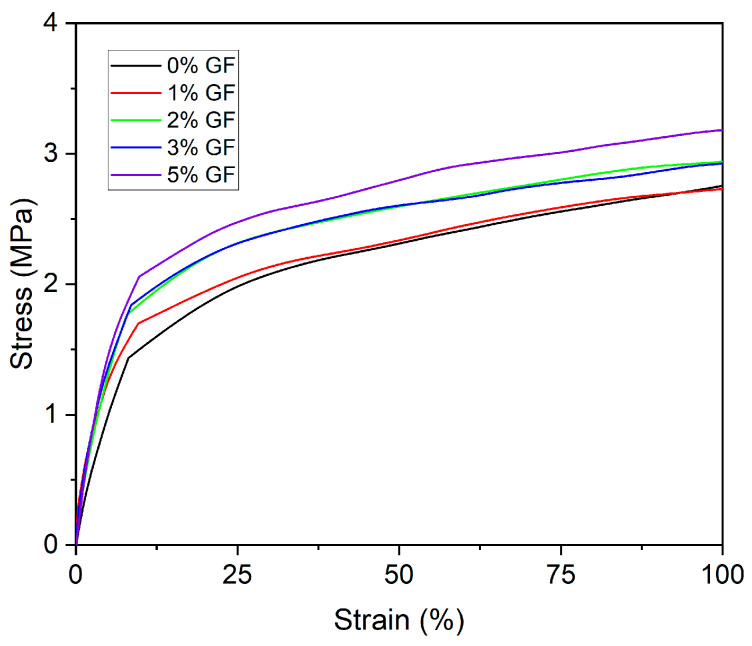
“σ-ε” curves for PIM-0%GF–PIM-5%GF. All PIMs contained PVDF-HFP: Aliquat 336 mass ratio of 6:4. Experimental conditions: tests were carried out at room temperature (25 ± 1 °C) and at crosshead speed of 1 mm min^−1^ for tensile modulus and 5 mm min^−1^ as test speed. All PIMs contained PVDF-HFP:Aliquat 336 mass ratio of 6:4.

**Figure 5:**
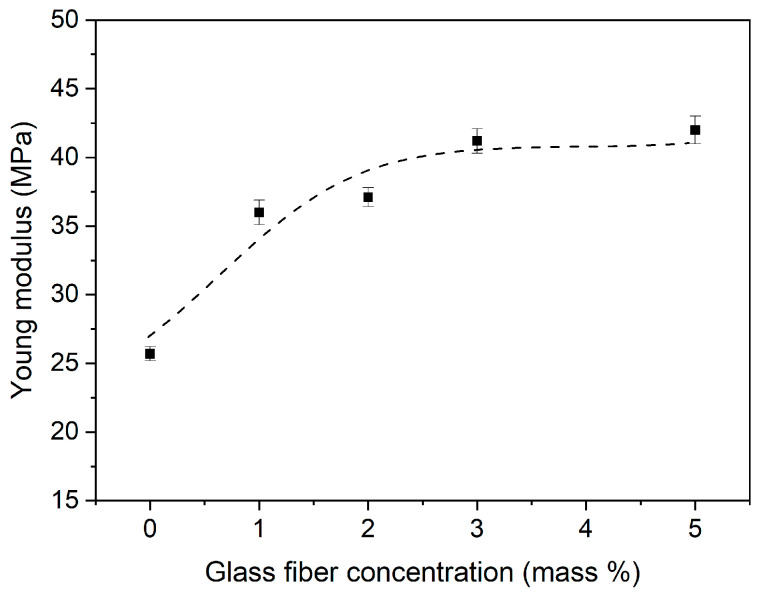
Young modulus vs. glass fiber concentration in PIMs containing PVDF-HFP:Aliquat 336 mass ratio of 6:4. Experimental conditions are as stated in Figure 4.

The mechanical properties of PIM-0%GFs–PIM-5%GF were characterized by mechanical tensile tests, and the corresponding “stress–strain” curves are shown in Figure 4. The elastic (Young) modulus of the membranes was found to increase with increasing the GF concentration from about 25 MPa for PIM-0%GF to 40 MPa for PIM-5%GF. However, this increase was found to be most pronounced between 2 and 3% (Figure 5). The dependence of the tensile strength with increasing GF concentration followed a similar trend (Figure 4). In terms of plasticity, this dependence was in the opposite direction, i.e., it decreased with increasing the GF concentration. It was concluded that an acceptable compromise between strength and plasticity was achieved for PIM-3%GF. At such GF concentration, this PIM exhibited improved mechanical strength compared to the conventional PIM-0%GF, without a significant deterioration in its plasticity.

**Figure 6:**
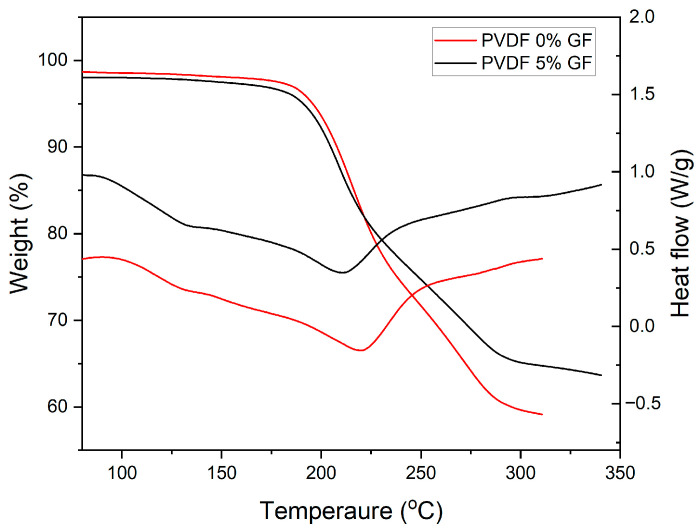
TGA and DTA curves of PIM-0%GF and PIM-5%GF. All PIMs contained PVDF-HFP:Aliquat 336 mass ratio of 6:4. Experimental conditions: thermal behavior of the membranes was investigated at 10 K/min under pure nitrogen atmosphere.

The TGA curves of the PIMs with GFs show better retention of the carrier Aliquat 336 at elevated temperatures compared to the PIM without GFs, as illustrated in Figure 6, which shows the TGA and DTA curves of PIM-0%GF and PIM-5%GF. This effect might be the result of increased tortuosity of the membrane structure due to the presence of GFs, thus hindering the diffusion of Aliquat 336 towards the membrane surface. The good adhesion between the GFs and the PVDF-HFP/Aliquat 336 part of the PIM, discussed earlier (Figure 2), could be another reason for the better retention of the carrier at elevated temperatures.

The improved mechanical strength of PIMs with embedded GFs can be explained by the fact that the fibers reinforce the membranes’ structure, thus reducing the mobility of the polymer chains and limiting deformation under mechanical stress.

### 3.2. Transport and Stability Properties of the PIMs

The transport properties of PIM-0%GF–PIM-5%GF were evaluated using the thiocyanate ion as a model target chemical species. This ion is present in mine tailings waters and its removal from such waters has been successfully achieved by a PIM-based clean-up approach [13].

The transport of SCN_−_ across the PIMs studied is a typical example of facilitated transport [1,64,65] and it can be considered as consisting of three main steps, assuming that both the feed and receiving solutions are ideally mixed. In the first step, SCN_−_ is extracted from the feed solution into the PIM as a result of an interfacial ion-exchange reaction (Equation (2)). This is followed by the diffusion of the ion pair (NR_4_)^+^SCN^−^ within the PIM towards the PIM/receiving solution interface. At this interface SCN_−_ is back-extracted into the receiving solution as a result of the ion-exchange reaction described by Equation (2), but in this case the equilibrium is shifted to the left. This results in the regeneration of the carrier ion pair ((NR_4_)^+^NO_3_^−^), which diffuses back to the PIM/feed solution interface to transport another SCN^−^ anion from the feed to the receiving solution. The driving force in this facilitated transport process is the high concentration of the back-extracting reagent, i.e., 1 mol L^−1^ NO_3_^−^.

(2) $${\left[{SCN}^{-}\right]}_{aq}+{\left[{\left(NR_{4}\right)}^{+}{NO}_{3}^{-}\right]}_{PIM}\Leftrightarrow {\left[{\left(NR_{4}\right)}^{+}{SCN}^{-}\right]}_{PIM}+{\left[{NO}_{3}^{-}\right]}_{aq}$$

**Figure 7:**
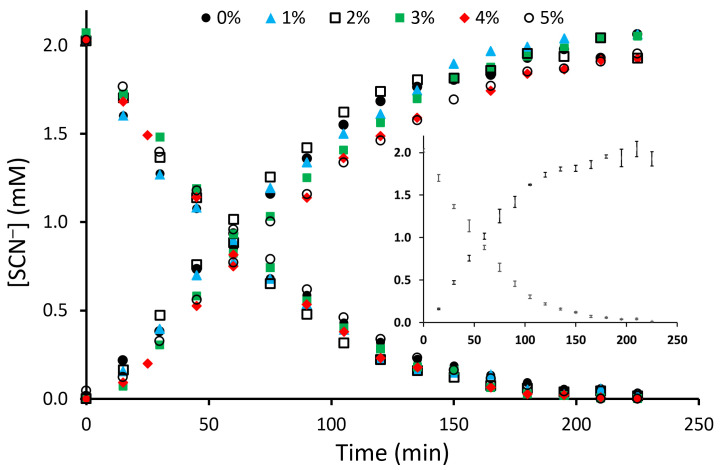
Extraction and back-extraction curves in the SCN_−_ transport experiments across PIM-0%GF–PIM-5%GF. The markers referring to PIMs with different GF concentrations are defined in the figure. Experimental conditions: feed solution—120 mL, 2 mM SCN_−_; receiving solution—120 mL, 1 M NaNO_3_; stirring rate—600 rpm of both solutions; PIM—6:4 mass ratio of PVDF-HFP: Aliquat 336; inset: errors bars for the PIM-2%GF transport experiment illustrating experiment repeatability.

Extraction and back-extraction curves for the PIMs with or without glass fibers, obtained in the corresponding transport experiments, are presented in Figure 7. Since the interfacial ion-exchange and transmembrane transport processes did not involve the participation of hydrogen or hydroxide ions, the pH of the feed (pH_t=0 h_ = 6.19 ± 0.09; pH_t=6 h_ = 6.34 ± 0.07) and receiving (pH_t=0 h_ = 6.63 ± 0.18; pH_t=6 h_ = 6.31 ± 0.09) solutions changed only insignificantly. The slightly higher pH_t=0 h_ value of the feed solution can be explained by the fact that NaSCN is the salt of a strong base (NaOH) and a moderately strong acid (HSCN, K_a_ = 0.13 [66]) and therefore hydrolyses to a small extent.

The following equation has been used frequently to describe transmembrane transport [1]:

(3) $$ln\left(C/C_{0}\right)=-\;\left(A/V\right)Pt$$

where *C*_0_ and *C* are the initial and transient concentrations of the transported chemical species (SCN) in the feed solution in mol m^−3^; *t* is time in s; *A* is the membrane surface area exposed to the feed solution in m^2^, *V* is the volume of the feed solution in m^3^; and *P* is permeability.

The permeability values for the PIM-0%GF—PIM-5%GF were calculated (Table 1) by fitting Equation(3) to the corresponding extraction data for the first 90 min of the transport process (Figure 7). Reasonably good fits were obtained in all cases with R^2^ values in the range of 0.986–0.996. The *P* values were used to calculate the initial flux (*J*_0_) (Table 1) using Equation (4) [1]:

(4) $$J_{0}=-V/A{\left(dC/dt\right)}_{t=0}=P\;C_{0}$$

The permeability and initial flux values were similar in value for PIM-0%GF–PIM-4%GF and only slightly lower for PIM-5%GF, thus indicating that glass fibers did not obstruct thiocyanate transport up to a GF concentration of 4%.

**Figure 8:**
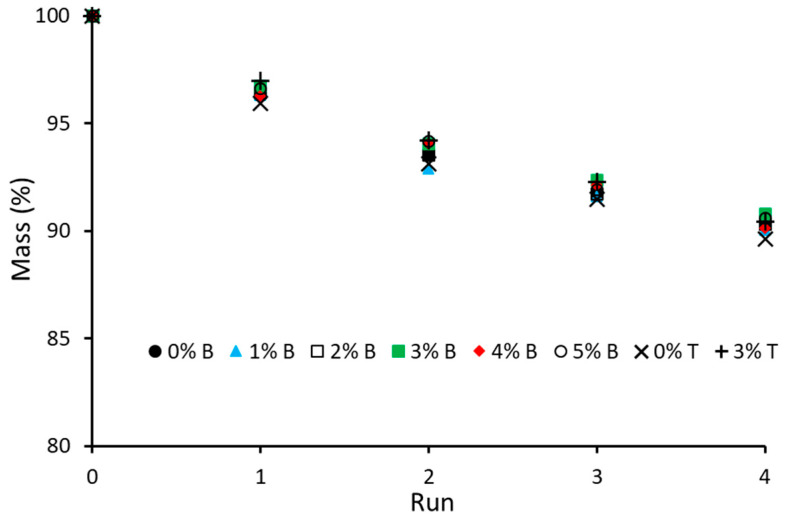
PIM-0%GF–PIM-5%GF mass change due to Aliquat 336 leaching into the aqueous phase(s) in the batch (B) and transport (T) experiments. All PIMs contained PVDF-HFP:Aliquat 336 mass ratio of 6:4.

The results of the Aliquat 336 batch leaching experiments of PIM-0%GF–PIM-5%GF in 0.1 M NaCl solutions showed that the GFs did not affect the loss of Aliquat 336 into the aqueous solution (Figure 8).

**Figure 9:**
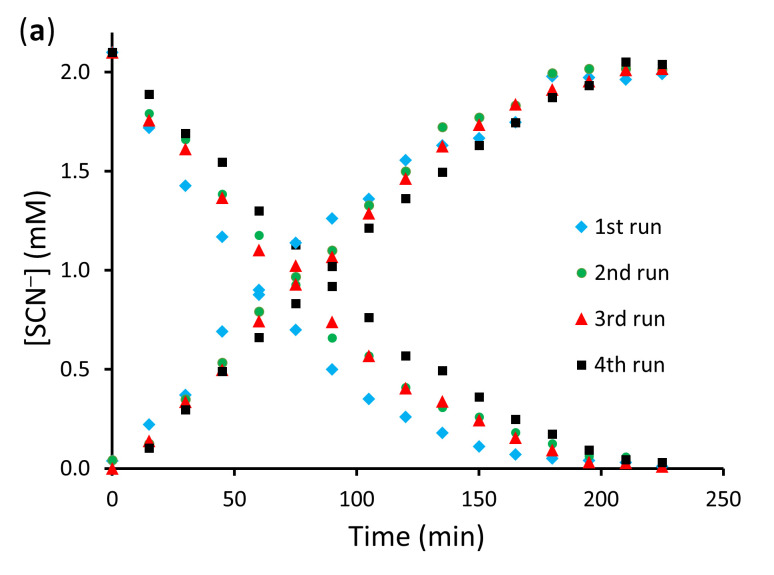

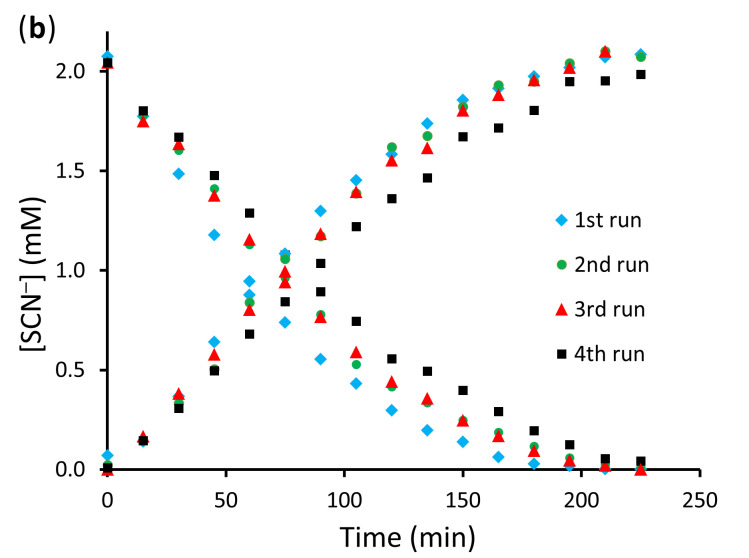
Extraction and back-extraction curves in SCN_−_ transport across PIM-0%GF (**a**) and PIM-3%GF (**b**) in 4 consecutive transport experiments. Remaining experimental conditions are as stated in Figure 7.

The stability of PIM-3%GF, which provided an acceptable compromise between strength and plasticity, and that of the conventional PIM-0%GF were compared in four consecutive transport experiments. The resulting extraction and back-extraction curves (Figure 9) indicated similar stability, irrespective of the presence or absence of GFs. This result could be explained with the similar loss of Aliquat 336 from the two PIMs which closely resembled the results of the batch leaching experiments (Figure 8).

## 4. Conclusions

GFs were introduced successfully in PIMs containing PVDF-HFP as the base polymer and Aliquat 336 as the carrier in a mass ratio of 6:4 and were found to distribute relatively uniformly within the PIM structures in concentrations up to 5%. The GFs did not change the amorphous character of the PIMs and were found to improve the retention of the carrier at elevated temperatures. The GF-containing PIMs exhibited higher elastic moduli and tensile strength than their counterparts without GFs, with both properties increasing with increasing the fiber concentration. The transport properties of PIMs with and without GFs were compared using thiocyanate as a model target chemical species. The PIMs without or with GFs exhibited similar loss of Aliquat 336 in four consecutive batch leaching experiments and similar transport properties, irrespective of the GF concentration. The fibers did not affect the long-term stability of PIM-3%GF, which provided an acceptable compromise between strength and plasticity. On the basis of the results obtained, it can be concluded that the incorporation of GFs with a concentration of 3 or 4% in PVDF-HFP/Aliquat 336 PIMs has improved their mechanical properties without negatively impacting on the membrane transport properties and long-term stability, thus improving PIM suitability for industrial applications where both mechanical robustness and efficient ion extraction/transport performance are required. It should be pointed out that in the potential future adoption of the PIMs studied in practical applications, it would be advisable to use less toxic and more environmentally friendly solvents than THF, such as 2-methyltetrahydrofuran and ethyl acetate [67].

## Figures and Tables

**Table 1:** Permeability and initial flux values of PIM-0%GF–PIM-5%GF.

GF Concentration (%)	0	1	2	3	4	5
10^5^ × *P* (m s^−1^)	1.46 ± 0.04	1.52 ± 0.04	1.52 ± 0.05	1.44 ± 0.05	1.48 ± 0.06	1.30 ± 0.04
10^5^ × *J*_0_ (mol m^−2^ s^−1^)	2.96 ± 0.08	3.09 ± 0.08	3.09 ± 0.09	3.43 ± 0.12	2.90 ± 0.10	2.64 ± 0.11

## Data Availability

The original contributions presented in this study are included in the article. Further inquiries can be directed to the corresponding authors.
